# Case Report: A combination of *CHEK2* and high polygenic risk score leads to early-onset male breast cancer

**DOI:** 10.3389/fonc.2026.1764722

**Published:** 2026-03-16

**Authors:** Kamil Tamindarov, Marika Frank, Sonja Wegscheider, Beatrix Benedix, Thomas Lingscheidt, Kai Neukirchner, Hadeel Shamma, Jens Schnabel, Marion Tolkmitt, Karina Pillau, Vincent Strehlow, Julia Hentschel, Diana Le Duc

**Affiliations:** 1 Faculty of Medicine Hospital Carl Gustav Carus, TU Dresden, Dresden, Germany; 2 Institute for Clinical Genetics, University Hospital Carl Gustav Carus at TUD Dresden University of Technology and Faculty of Medicine of TUD Dresden University of Technology, Dresden, Germany; 3 Center for Diagnostics, Human Genetics Department, Chemnitz Clinics, Chemnitz, Germany; 4 Institute of Human Genetics, University of Leipzig Medical Center, Leipzig, Germany; 5 DRK Hospital Chemnitz Rabenstein, Women’s Hospital, Chemnitz, Germany; 6 MVZ Diagnosticum GmbH, Stollberg-Niederdorf, Germany; 7 Institute for Pathology, Chemnitz Clinics, Chemnitz, Germany; 8 ERN GENTURIS, Hereditary Cancer Syndrome Center Dresden, Dresden, Germany; 9 National Center for Tumor Diseases (NCT), NCT/UCC Dresden, a partnership between DKFZ, Faculty of Medicine and University Hospital Carl Gustav Carus, TUD Dresden University of Technology, and Helmholtz-Zentrum Dresden-Rossendorf (HZDR), Dresden, Germany; 10 German Cancer Consortium (DKTK), partner site: Dresden, and German Cancer Research Center (DKFZ), Heidelberg, Germany

**Keywords:** case report, *CHEK2*, male breast cancer, MSH6, polygenic risk score

## Abstract

Male breast cancer (MBC) is a rare disease, accounting for about 1% of all breast cancer cases worldwide. Compared to female breast cancer (FBC), MBC shows a higher prevalence of hormone receptor positivity and distinct germline predispositions, most frequently pathogenic variants in *BRCA2* and *CHEK2*. The contribution of mismatch repair (MMR) genes such as *MSH6* to MBC risk remains, however, unclear. In addition, polygenic risk scores (PRS) have emerged as promising tools for breast cancer risk prediction but are not yet used in routine care. We report the case of a 42-year-old man diagnosed with invasive carcinoma of no special type, strongly estrogen receptor–positive, HER2-negative, and with a family history of breast and prostate cancer. Genetic testing revealed a pathogenic *CHEK2* nonsense variant (p.Trp411*), a likely pathogenic *MSH6* frameshift variant, and a breast cancer PRS in the 99th percentile. Tumor sequencing confirmed both germline variants but showed microsatellite stability and no loss of heterozygosity, arguing against a causal role of *MSH6*. This case illustrates how PRS, in combination with moderate-risk variants, like those in *CHEK2*, may drive early-onset MBC and highlights the need to incorporate polygenic models into risk assessment and counseling.

## Introduction

Breast cancer is predominantly a disease of women, yet in rare occurrences it also affects males, accounting for approximately 1% of breast cancer cases worldwide (1, 2). Due to its rarity, male breast cancer (MBC) is often diagnosed at a later stage, as awareness among both patients and health care providers is limited. In general, treatment and diagnostic strategies are extrapolated from guidelines for female breast cancer (FBC). However, despite similarities with FBC, important differences may exist in presentation and biology. E.g. MBC is more likely to be hormone receptor positive (estrogen (ER+) and progesterone (PR+)), than FBC (1, 3, 4). In some studies, more than 95% of MBC cases are ER-positive, rendering MBC a more homogeneous group than FBC (1, 4). Additionally, FBC appears to also have a more diverse genetic basis compared to MBC (4). The germline genetic variants differ between male and female breast cancer primarily in the prevalence and spectrum of high-penetrance mutations, especially in *BRCA* genes. Pathogenic variants in *BRCA2* are markedly more common in MBC than in FBC, with *BRCA2* accounting for the majority of hereditary cases in men, while *BRCA1* is more frequently implicated in women (5). Moderate penetrance genes such as *CHEK2* contribute to risk in both sexes, but their relative impact is considered to be less pronounced in men. *CHEK2* pathogenic variants account for about 2–3% of the all MBC (6, 7, 9); most of these cases are associated with the pathogenic *CHEK2*-variant c.1100delC, which leads to a loss-of-function (LoF). Female mutation carriers have a lifetime risk of breast cancer of approximately 20–30% (4, 8). Although the absolute risk is significantly lower in men, a 3.7-fold increase in risk compared to the general population has been observed (6, 9). In women, additional genes beyond *BRCA1* and *BRCA2* have been associated with breast cancer susceptibility. Notably, DNA mismatch repair (MMR) genes, especially *MSH6*, have been implicated in conferring a modestly increased risk. Breast cancers arising in the context of Lynch syndrome are often hormone receptor–positive and may exhibit mismatch repair deficiency (MMR-D) and microsatellite instability, particularly in carriers of *MSH6* pathogenic variants (10, 11). However, there is currently no clear evidence for a significant association between germline *MSH6* pathogenic variants and MBC disease risk. Large sequencing studies in MBC cohorts have not yet identified *MSH6* as a major contributor to risk in men, and the prevalence of pathogenic *MSH6* variants in male breast cancer is very low or absent (7, 12, 13).

Polygenic burden is another genetic risk factor for breast cancer in both sexes. Genome-wide association studies show that common low-penetrance susceptibility loci largely overlap between sexes, but some single nucleotide polymorphisms (SNPs) may exhibit gender-specific effect sizes (14–16). Polygenic risk scores (PRS) can provide meaningful risk stratification for MBC, however this is not part of the standard clinical management (7, 15, 17–19). Here, we present the case of a 42-year-old male with breast cancer. Genetic analysis revealed a pathogenic variant in *CHEK2*, another pathogenic variant in *MSH6*, and a PRS in the 99th percentile (or 0.99 quantile). To better understand what the causative variant is, we further analyzed tumor tissue. We argue that PRS should be incorporated in routine clinical practice, alongside with known genes for tumor predisposition syndromes.

## Case description

**Figure 1:**
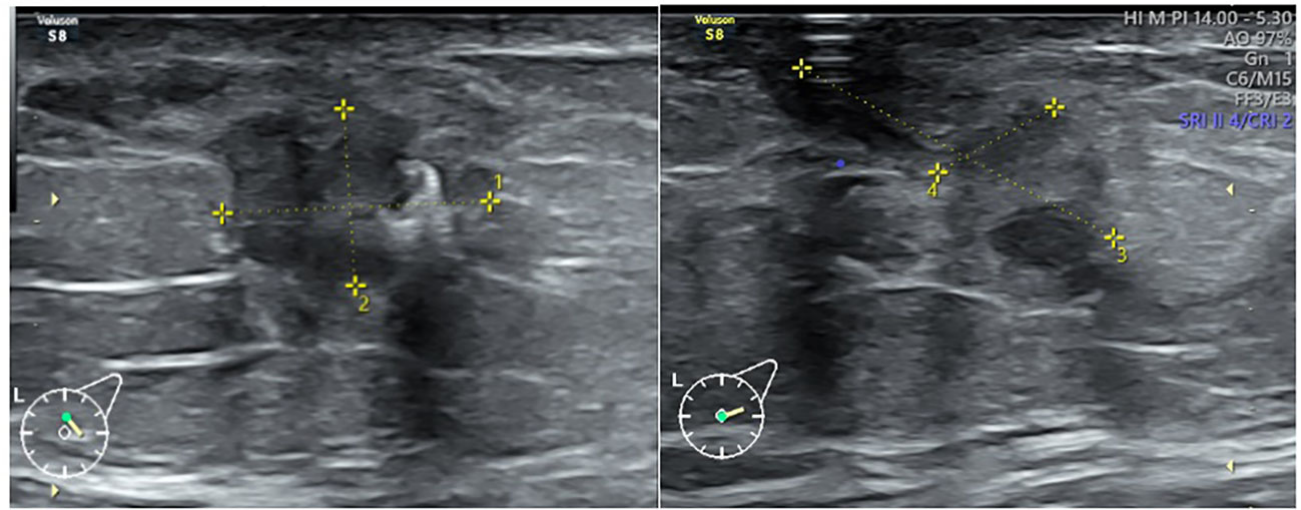
Ultrasound images of the left breast from two different angles. The predominantly hypoechoic mass lesion with irregular shape is visible. Maximum dimensions measured were 13.15 mm (1), 8.50 mm (2), 18 mm (3), and 6.77 mm (4).

A 42-year-old male patient was admitted with swelling of the left breast, where he had noticed a palpable mass. On physical examination, a firm, immobile mass was detected in the left breast, whereas the contralateral breast was unremarkable. The patient’s BMI was 26.9 kg/m^2^. The patient family history was notable in respect to his sister who had died from breast cancer at the age of 39 years and his father who had been diagnosed with prostate carcinoma, aged 77. The patient’s personal medical history was otherwise unremarkable. Breast ultrasound revealed an irregularly shaped, ill-defined lesion located in the retroareolar region of the left breast at 3–4 o’clock, measuring approximately 1,3 × 0,8 × 1,8 cm (**Figure 1**). The lesion extended up to the skin and demonstrated prominent vascularization on ultrasound. According to the German adaptation of the BI-RADS classification (Madjar) for ultrasound, the lesion was categorized as BI-RADS 6. The right breast showed no pathological findings, and the axillary lymph nodes were without pathology. A punch biopsy of the lesion was obtained for histopathological analysis, and a marker clip was placed in the lesion.

To exclude distant metastases, computed tomography (CT) and whole-body bone scintigraphy were performed. CT confirmed a small firm lesion of approximately 7 mm in the left breast with the biopsy clip *in situ*, consistent with the ultrasound findings. No suspicious lymph nodes were identified. Whole-body bone scintigraphy and SPECT/CT showed no evidence of metastatic disease. The lesion was classified as cT1c, cN0, cM0, Stage I, along with the ultrasound results.

**Figure 2:**
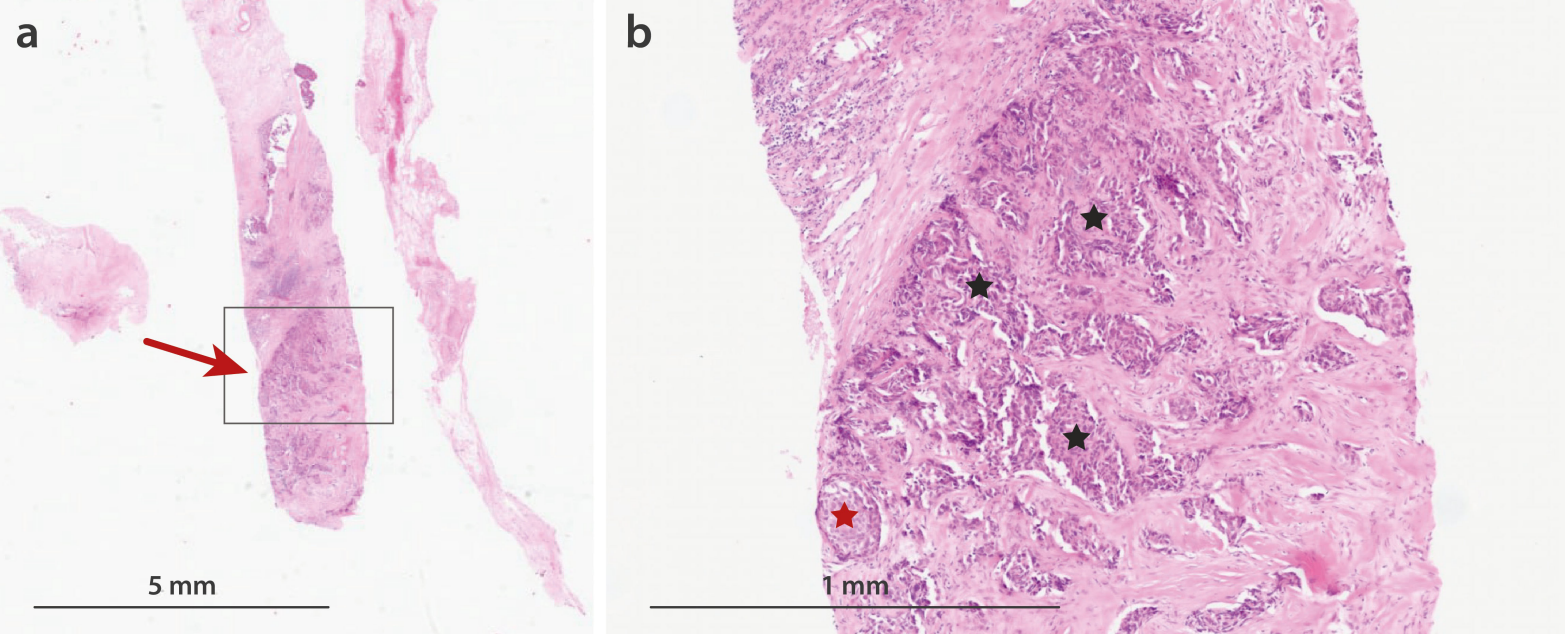
Histopathological analysis of the left breast punch biopsy in 2 magnifications. **(a)** Hematoxylin and eosin (H&E) staining under low magnification (10×) showing the overall tissue architecture. The arrow indicates the tumor tissue. **(b)** Medium magnification (100×) demonstrating moderately differentiated invasive carcinoma of no special type (NST, G2). Black Stars mark indicate invasive parts. Red star marks DCIS G2. To evaluate the potential role of MSH6 in tumor formation, microsatellite analysis and molecular genetic testing of the tumor tissue were performed. For this, the Oncimine Comprehensive Assay v3 on the platform Ion GeneStudio S5 (Thermo Fisher Scientific) was used, which covers 86 hotspot genes, the full exonic sequence of 48 genes, and copy number changes in 43 genes (see **Supplementary Table 2** in the **Supplementary Material**). These analyses revealed the same pathogenic variants as previously identified in the germline, thereby validating the prior findings. The allele frequencies of MSH6 and CHEK2 were 54% and 67%, respectively, consistent with the absence of loss of heterozygosity. The estimated tumor cell content was 70%. No pathogenic variants were detected in BRCA1, BRCA2, or ERBB2. All seven markers of microsatellite instability were stable. Immunohistochemistry for mismatch repair proteins was not performed.

Histopathological analysis of the punch biopsy revealed invasive carcinoma of no special type (NST) (**Figures 2a, b**) with associated ductal carcinoma *in situ* (DCIS, grade 2). Immunohistochemical staining demonstrated strong estrogen receptor expression (immunoreactive score (IRS) 12 points), weak progesterone receptor expression (IRS 4 points), a Ki-67 proliferation index of 28%, and HER2 expression was equivocal (score +1).

Genomic DNA was extracted from the proband’s peripheral blood to perform cancer panel sequencing (TWIST Custom panel, TE-96574338), targeting 219 genes (see **Supplementary Table 1** in the Supplementary Material), using next-generation sequencing (NGS) with the Twist Library Preparation EF Kit and the Twist Universal Adapter System. A heterozygous nonsense germline variant chr22:28,695,737; NM_007194.4, c.1232G>A, (pTrp411*) in *CHEK2* was identified which was classified as pathogenic (1, 9, 20). Additionally, another likely pathogenic heterozygous variant, chr2:47,800,788; NM_000179.3: c.2805del, p. (Asp939Ilefs*9), was detected in *MSH6 (*21).

**Figure 3:**
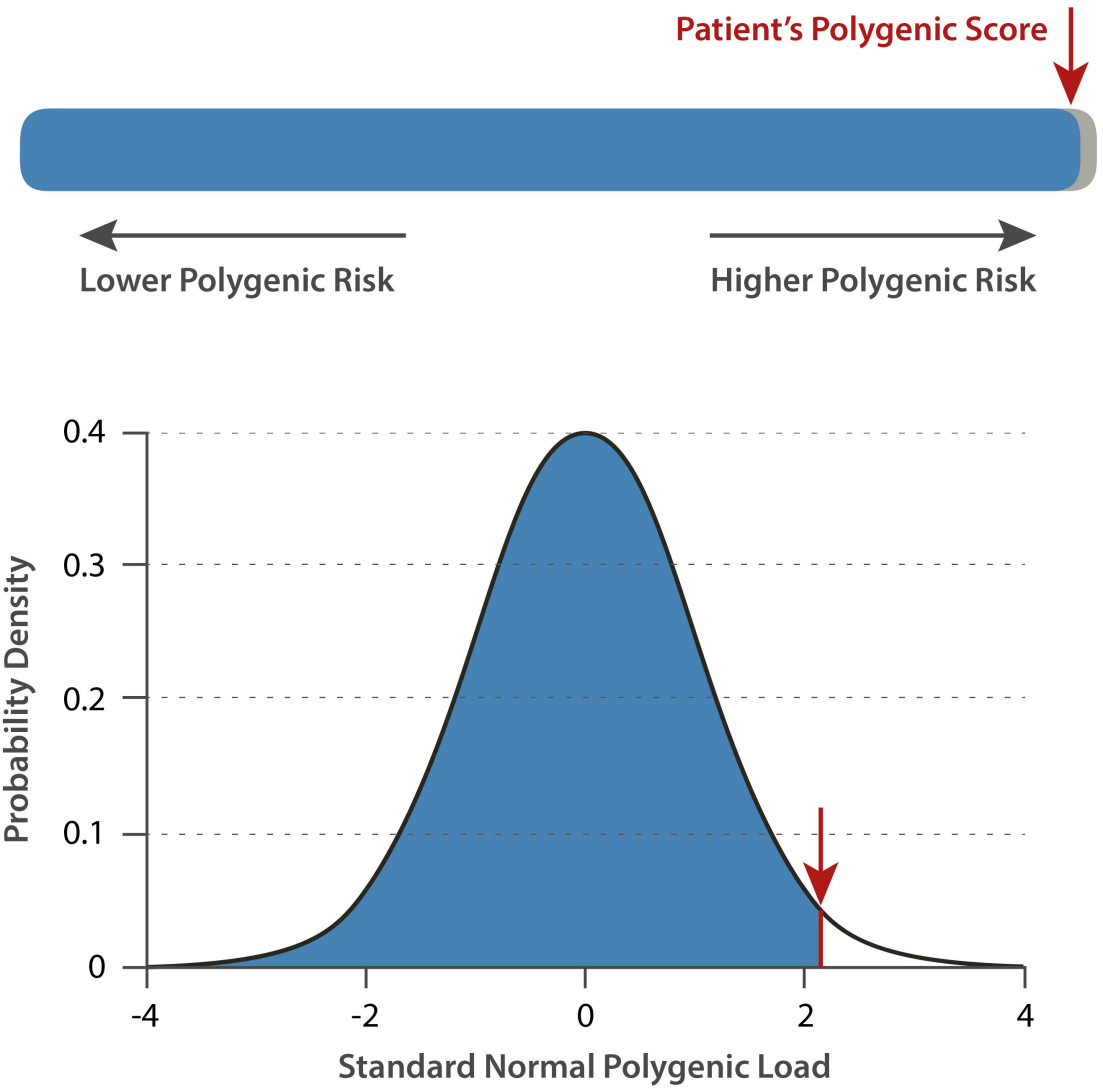
The distribution of the PRS in the population. The red line indicates the position of the patient within the distribution of the patient compared to the general population (Figure is generated using the CanRisk web tool (https://canrisk.org, accessed 09.2025, modified)).

The polygenic risk score (PRS) for breast cancer was calculated as 2.173z^18^, corresponding to the 99th percentile (**Figure 3**), which is considered indicative of an increased predisposition to tumor development. The calculation of PRS was based on 313 breast cancer-associated variants (BCAC313) as defined by Mavaddat et al., 2019 (14). The use of the BCAC313 PRS model has so far only been validated for individuals of European genetic ancestry (14). Single nucleotide polymorphisms (SNPs), which cannot be covered with probes or called in an appropriate way, were imputed according to the recommendations of the German Consortium for Hereditary Breast and Ovarian Cancer (22).

**Figure 4:**
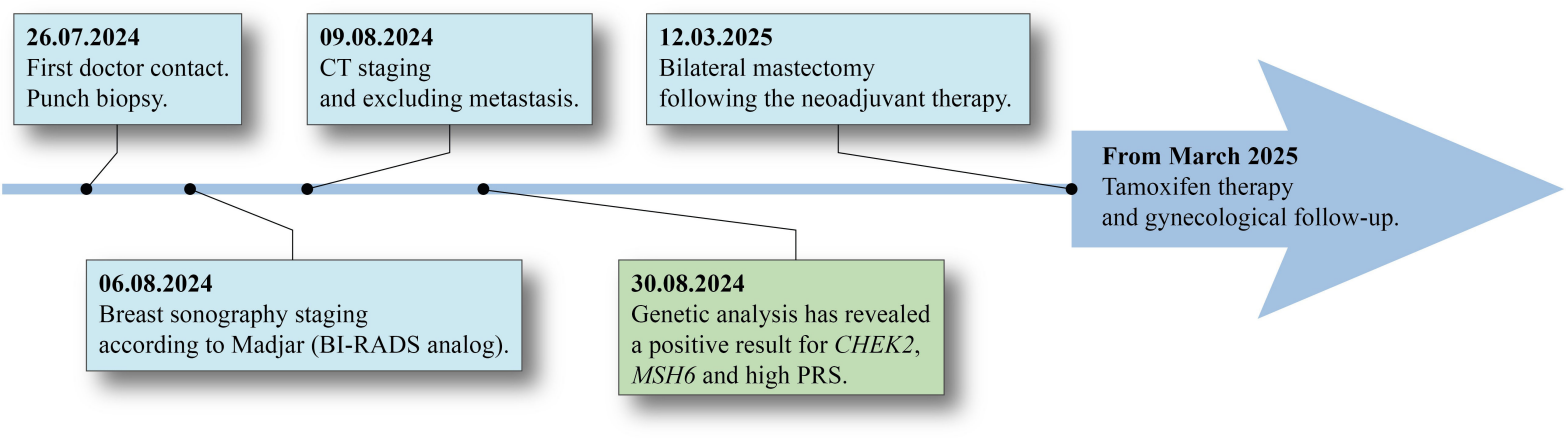
Patient history: From the initial physical examination through to the operation and subsequent follow-up.

To avoid a recurrence or secondary tumor developing on the right side, the patient opted for a bilateral mastectomy following neoadjuvant chemotherapy with epirubicin, cyclophosphamide, and paclitaxel. Postoperative histopathological analysis revealed signs of tumor regression as well as an additional tumor focus measuring 6 mm, consistent with invasive carcinoma of no special type (NST). Sentinel lymph nodes were free of tumor infiltration, and no evidence of perineural invasion was observed. The TNM classification of the tumor was ypTb, pN0(sn), cM0. The proliferative index (MIB-1/Ki-67) had decreased to 4%. The right breast had no sign of malignancy. Postoperatively, the patient received tamoxifen therapy and attended regular follow-up examinations with the gynecologist approximately every three months (**Figure 4**).

## Discussion

We describe a male patient carrying pathogenic germline variants in *CHEK2* and *MSH6* who developed breast cancer at an early age. Among the known risk factors for MBC, the pathogenic germline variant in *CHEK2*, family history, and overweight were positive in this patient. Protein-truncating *CHEK2* variants are generally associated with moderate risk for MBC with substantial heterogeneity depending on the studied population and variant (1, 6, 23). The initial diagnosis for the current patient occurred 12 years earlier than the average age of MBC associated with *CHEK2* mutations (6). Notably, due to heightened vigilance stemming from his family history, the patient performed self-examinations, which likely contributed to early detection at stage I. In general, men are often diagnosed with breast cancer later than women, frequently presenting with more advanced and aggressive disease (1, 2). Because criteria for genetic testing were not met at the time of the diagnosis for the patient’s sister, no genetic data of the sister is available.

In the context of Lynch syndrome, breast cancer may arise as an extracolonic malignancy. Among women with Lynch syndrome, up to 33% of breast cancers have been reported in association with *MSH6* mutations. By contrast, male breast cancer has not yet been reported in Lynch syndrome carriers (10, 24, 25). The current evidence does not support a causal link between *MSH6* mutations and MBC. While some studies have reported an increased incidence of breast cancer in female *MSH6* carriers, the association is modest and primarily observed in women, with standard incidence ratios around 2.1, and is not specific to male breast cancer (26). In our patient, the tumor was microsatellite stable and retained heterozygosity for *MSH6*, suggesting that the likely pathogenic variant in this gene did not play a role in tumorigenesis.

Importantly, the patient exhibited a high PRS, within the 99th percentile, in combination with a *CHEK2* truncating pathogenic variant. This combination may have had a crucial impact on tumor formation. High PRS can accelerate the accumulation of somatic mutations, akin to water gradually filling a glass until it overflows (18). The preservation of heterozygosity in the tumor further supports a multifactorial model of cancer development, where the cumulative effect of multiple low- to moderate-risk alleles contributes to early disease onset, rather than a single high-penetrance mutation. This observation aligns with emerging evidence on the polygenic contributions to carcinogenesis.

This case provides additional insight into the mechanisms underlying MBC and highlights the potential utility of PRS calculation in risk assessment, early detection, and personalized management. However, PRS is not yet standard of care for MBC risk assessment or management, as noted by the American College of Medical Genetics and Genomics, but is expected to be incorporated in future practice as tools and evidence mature (6, 19).

In conclusion, PRS testing may become a valuable tool for MBC research and clinical practice, enabling earlier diagnosis, risk stratification, and targeted surveillance. Further studies are needed to determine how PRS can be effectively integrated into routine care, particularly for male patients, for whom breast cancer remains rare but clinically significant.

## Conclusion

Male breast cancer remains rare, yet its genetic architecture increasingly mirrors that of female breast cancer, with a substantial proportion of risk potentially explained by the combined effect of moderate-penetrance genes and polygenic burden rather than high-penetrance mutations alone. Integration of polygenic risk scores into clinical risk assessment, alongside panel-based germline testing and, when needed, tumor sequencing, offers a better understanding of individual risk and can guide personalized management and family counseling. Larger prospective studies are needed to refine PRS thresholds for male breast cancer and to establish evidence-based surveillance strategies for unaffected male carriers of moderate-risk variants. Until then, this case reinforces the value of comprehensive genetic evaluation, including polygenic risk, in men with breast cancer or strong family history, and emphasizes the need for heightened awareness that breast cancer is not exclusively a female disease.

## Patient perspective

The patient described the discovery of the lump in his left breast as a moment of profound shock: “I was showering felt something hard right behind the nipple. I knew immediately that this could be serious because of what happened to my sister.” Because his sister had died of breast cancer at age 39, he had been performing monthly self-examinations. He contacted his general practitioner soon after and was referred without delay to a breast center. He repeatedly stressed how crucial this prior knowledge and vigilance had been: “Without my sister’s history I would probably have ignored it for months and thought it was just some harmless swelling.”

The diagnosis itself was a heavy blow, but the early stage gave him hope from the outset. The genetic results were discussed with him in detail. He accepted the pathogenic *CHEK2* variant as “almost expected” given the family history of breast cancer, but the additional likely pathogenic *MSH6* variant initially caused significant worry about Lynch syndrome and further cancer risks for himself and his family. The extremely high polygenic risk score in the 99^th^ percentile finally provided him with an explanation for the unusually early onset: “That number explained better, why it happened to me so young even though *CHEK2* alone is only moderate risk.”

Neoadjuvant chemotherapy was physically and emotionally demanding. He described fatigue and especially the period of uncertainty while waiting for the pathologic response as the hardest part. He tolerated the subsequent bilateral mastectomy very well and decided on the bilateral procedure without hesitation: “After losing my sister and going through chemotherapy, I simply didn’t want to live with the constant fear that the right side could be next. Removing both breasts gave me peace of mind.” He was highly satisfied with the aesthetic result after reconstruction and quickly regained full quality of life.

A separate major concern arose regarding his then 10-year-old son, who had been receiving growth hormone therapy because of short stature. After the father’s genetic results became known, he immediately contacted the pediatric endocrinologist and human geneticist. Although his son is a minor, testing was performed with parental consent to guide further management of the growth hormone therapy. The boy was found to carry the same pathogenic *CHEK2* p.Trp411* variant. Although the evidence for an interaction between exogenous growth hormone and *CHEK2*-associated cancer risk is very limited and no clear guidelines exist, the perceived uncertainty caused considerable anxiety in the family. The endocrinologist offered intensified monitoring (abdominal and thyroid ultrasound every six months) as a precautionary measure, but after intensive family discussion and because height gain under therapy had been only minimal, the parents decided to discontinue growth hormone treatment altogether.

At the most recent follow-up, the patient reported being back to professional and physical activity and stated that, apart from the worry about his son, he now feels psychologically stable and “incredibly grateful” that the cancer was detected early and treated successfully.

## Data Availability

The original contributions presented in the study are included in the article/**Supplementary Material**. Further inquiries can be directed to the corresponding author.
