# Supplementary material for: Case Report: A combination of CHEK2 and high polygenic risk score leads to early-onset male breast cancer
Source: Front Oncol. 2026 Mar 16;16:1764722. doi: 10.3389/fonc.2026.1764722 (PMC13034568; doi:10.3389/fonc.2026.1764722)
Supplement: Supplementary file 1 [file Table1.docx]

**Supplementary Materials**

Table S1

| **The genes of the target sequences**  **(219)** | *ACD, AIP, ALK, AKT1, ANKRD26, APC, ATM, ATR, BAP1, BARD1, BLM, BMPR1A, BRAF, BRCA1, BRCA2, BRIP1, BUB1B, CASR, CBL, CDC73, CDH1, CDK4, CDKN1B, CDKN1C, CDKN2A, CDKN2B, CEBPA, CHEK2, CIC1, CTR9, CTRC, DDB2, DDX41, DICER1, DIS3L2, DKC1, DOCK8, ELANE, EPCAM, ERCC1, ERCC2, ERCC3, ERCC4, ERCC5, ETV6, EXT1, EXT2, EZH2, FANCA, FANCB, FANCC, FANCD2, FANCE, FANCF, FANCG, FANCI, FANCL, FANCM, FAS, FH, FLCN, FOXE1, GALNT12, GATA1, GATA2, GBA, GPC3, HAX1, HOXB13, HRAS, ISCA.37401-loss, ITK, KIF1B, KIT, KRAS, LIG4, LZTR1, MAD2L2, MAP2K1, MAP2K2, MAX, MEN1, MET, MITF, MLH1, MRE11A, MSH2, MSH6, MTAP, MUTYH, NAF1, NBN, NF1, NF2, NHP2, NOP10, NRAS, NSD1, NTHL1, PALB2, PARN, PAX5, PDGFRA, PDGFRB, PHOX2B, PIK3CA, PMS2, POLD1, POLE, POLH, POT1, PPP1CB, PRF1, PRKAR1A, PRSS1, PTCH1, PTEN, PTPN11, RAD50, RAD51, RAD51B, RAD51C, RAD51D, RAF1, RB1, RECQL4, REST, RET, RHBDF2, RINT1, RIT1, RMRP, RPL11, RPL15, RPL23, RPL26, RPL27, RPL31, RPL35A, RPL36, RPL5, RPS10, RPS15, RPS17, RPS19, RPS24, RPS26, RPS27, RPS27A, RPS28, RPS29, RPS7, RTEL1, RUNX1, SAMD9L, SBDS, SDHA, SDHAF2, SDHB, SDHC, SDHD, SEC23B, SH2B3, SH2D1A, SHOC2, SLC5A5, SLX4, SMAD4, SMARCA4, SMARCB1, SMARCE1, SOS1, SOS2, SPINK1, SPRED1, SQSTM1, STAT3, STK11, SIN1, STX11, STXBP2, SUFU, TERC, TERF2IP, TERT, TINF2, TMEM127, TP53, TRIM28, TRIM37, TRIP13, TSC1, TSC2, TSR2, UBE2T, UNC13D, VHL, WAS, WRAP53, WRN, WT1, XPA, XPC, XRCC2, KMT2D, FAT4, KMT2C, ATRX, RETN, STAT1, ARID1A, ARID1B, CTNNB1, IDH1, IDH2, GNA11, GNAQ, EGFR, MYD88.* |
| --- | --- |

Table S2

| **Hotspot Gene (86)** | *AKT1, AKT2, AKT3, ALK, AR, ARAF, AXL, BRAF, BTK, CBL, CCND1, CDK4, CDK6, CHEK2, CSF1R, CTNNB1, DDR2, EGFR, ERBB2, ERBB3, ERBB4, ERCC2, ESR1, EZH2, FGFR1, FGFR2, FGFR3, FGFR4, FLT3, FOXL2, GATA2, GNA11, GNAQ, GNAS, H3F3A, HIST1H3B, HNF1A, HRAS, IDH1, IDH2, JAK1, JAK2, JAK3, KDR, KIT, KNSTRN, KRAS, MAGOH, MAP2K1, MAP2K2, MAPK1, MAX, MDM4, MED12, MET, MTOR, MYC, MYCN, MYD88, NFE2L2, NRAS, NTRK1, NTRK2, NTRK3, PDGFRA, PDGFRB, PIK3CA, PIK3CB, PPP2R1A, PTPN11, RAC1, RAF1, RET, RHEB, RHOA, ROS1, SF3B1, SMAD4, SMO, SPOP, SRC, STAT3, TERT, TOP1, U2AF1, XPO1.* |
| --- | --- |
| **Full Exom (48)** | *ARID1A, ATM, ATR, ATRX, BAP1, BRCA1, BRCA2, CDK12, CDKN1B, CDKN2A, CDKN2B, CHEK1, CREBBP, FANCA, FANCD2, FANCI, FBXW7, MLH1, MRE11, MSH2, MSH6, NBN, NF1, NF2, NOTCH1, NOTCH2, NOTCH3, PALB2, PIK3R1, PMS2, POLE, PTCH1, PTEN, RAD50, RAD51, RAD51C, RAD51D, RAD51B, RB1, RNF43, SETD2, SLX4, SMARCA4, SMARCB1, STK11, TP53, TSC1, TSC2.* |
| **Copy number changes (43):** | *AKT1, AKT2, AKT3, ALK, AR, AXL, BRAF, CCND1, CCND2, CCND3, CCNE1, CDK2, CDK4, CDK6, EGFR, ERBB2, ESR1, FGF19, FGF3, FGFR1, FGFR2, FGFR3, FGFR4, FLT3, IGF1R, KIT, KRAS, MDM2, MDM4, MET, MYC, MYCL, MYCN, NTRK1, NTRK2, NTRK3, PDGFRA, PDGFRB, PIK3CA, PIK3CB, PPARG, RICTOR, TERT.* |
